# Luspatercept: A peaceful revolution in the standard of care for myelodysplastic neoplasms

**DOI:** 10.1002/hem3.41

**Published:** 2024-03-03

**Authors:** Francesca Vinchi, Uwe Platzbecker

**Affiliations:** ^1^ Iron Research Laboratory New York Blood Center New York New York USA; ^2^ Weill Cornell Medicine Department of Pathology and Laboratory Medicine New York New York USA; ^3^ Universitatsklinikum Leipzig Klinik und Poliklinik fur Hamatologie Zelltherapie und Hamostaseologie Leipzig Germany

Myelodysplastic syndromes/neoplasms (MDS) are a group of bone marrow failure disorders characterized by ineffective erythropoiesis and defective production of mature white blood cells and platelets, with a varying propensity of transformation to acute myeloid leukemia. Lower‐risk MDS (LR‐MDS) usually present with anemia and predisposition to frequent infections. In these patients, anemia is associated with fatigue, poor quality of life, and increased hospitalization and mortality. Chronic red blood cell (RBC) transfusions and/or erythropoietin (EPO)‐stimulating agents (ESAs; e.g., epoetin‐alfa or ‐beta) are often administered to correct the underlying anemia and to symptomatically manage MDS. However, patients who become RBC transfusion‐dependent have a significantly shorter overall survival than those who are transfusion‐independent, likely due to iron overload and/or a more severe underlying bone marrow disease.^1^ Moreover, about one‐third of MDS patients exhibit primary resistance to ESAs or lose response over time, about 2 years into treatment. Currently, the only potentially curative approach for MDS patients is allogeneic stem cell transplant, which is not accessible to all patients due to a lack of matching donors and/or significant treatment‐related morbidities and mortality.^1^ Therefore, finding valuable alternatives to transfusions and ESAs to correct anemia in MDS is a priority.

Recently, the erythroid maturation agent luspatercept has tremendously improved the ability to clinically manage anemia in MDS patients.

The clinical development of luspatercept was initiated after the incidental observation that a similar molecule, sotatercept, when administered for the treatment of osteoporosis in postmenopausal women, caused a significant increase in hemoglobin levels. Targeting activin receptor signaling was originally explored as a potential avenue to reduce bone loss in postmenopausal osteoporosis. However, the activin ligand trap sotatercept demonstrated an unexpected erythroid response in subjects with osteoporosis, which led to the exploration of this drug and its analogs in the amelioration of ineffective erythropoiesis.^2^

Luspatercept is a recombinant fusion protein consisting of a modified extracellular domain of the human activin receptor type IIB (ActRIIB) fused with the Fc domain of human immunoglobulin G1 (IgG1) (Figure 1). Unlike sotatercept, luspatercept binds only minimally to activin A and traps with high affinity the transforming growth factor‐β (TGF‐β) superfamily members to inhibit ActRIIB‐Smad2/3‐dependent signaling. Importantly, the activation of the Smad pathway interferes with late‐stage erythroid maturation. In preclinical studies, luspatercept has been shown to enhance late‐stage erythroid maturation, by inhibiting the GDF11‐mediated Smad2/3 signaling, both in steady‐state and stress conditions, regardless of EPO levels. While GDF11 was initially proposed as the major ligand of luspatercept, recent studies showing a lack of anemia improvement in the absence of GDF11 upon ineffective erythropoiesis suggest that other TGF‐β superfamily ligands are the functional targets of luspatercept. In MDS, excessive activation of the TGF‐β signaling has been reported. Luspatercept, by acting as ligand trap of the TGF‐β superfamily negative regulators of erythropoiesis, modulates TGF‐β signaling and promotes late‐stage maturation of erythroid precursors (Figure 1). Through these mechanisms, luspatercept alleviates ineffective erythropoiesis and ameliorates anemia in MDS patients.^2^ To date, the precise ligands and mode of action of luspatercept have not been completely clarified and require further research. Interestingly, luspatercept was demonstrated to directly modulate stromal cells to improve erythroid maturation^3^ and to mitigate bone loss in MDS vivo.^4^

**Figure 1:**
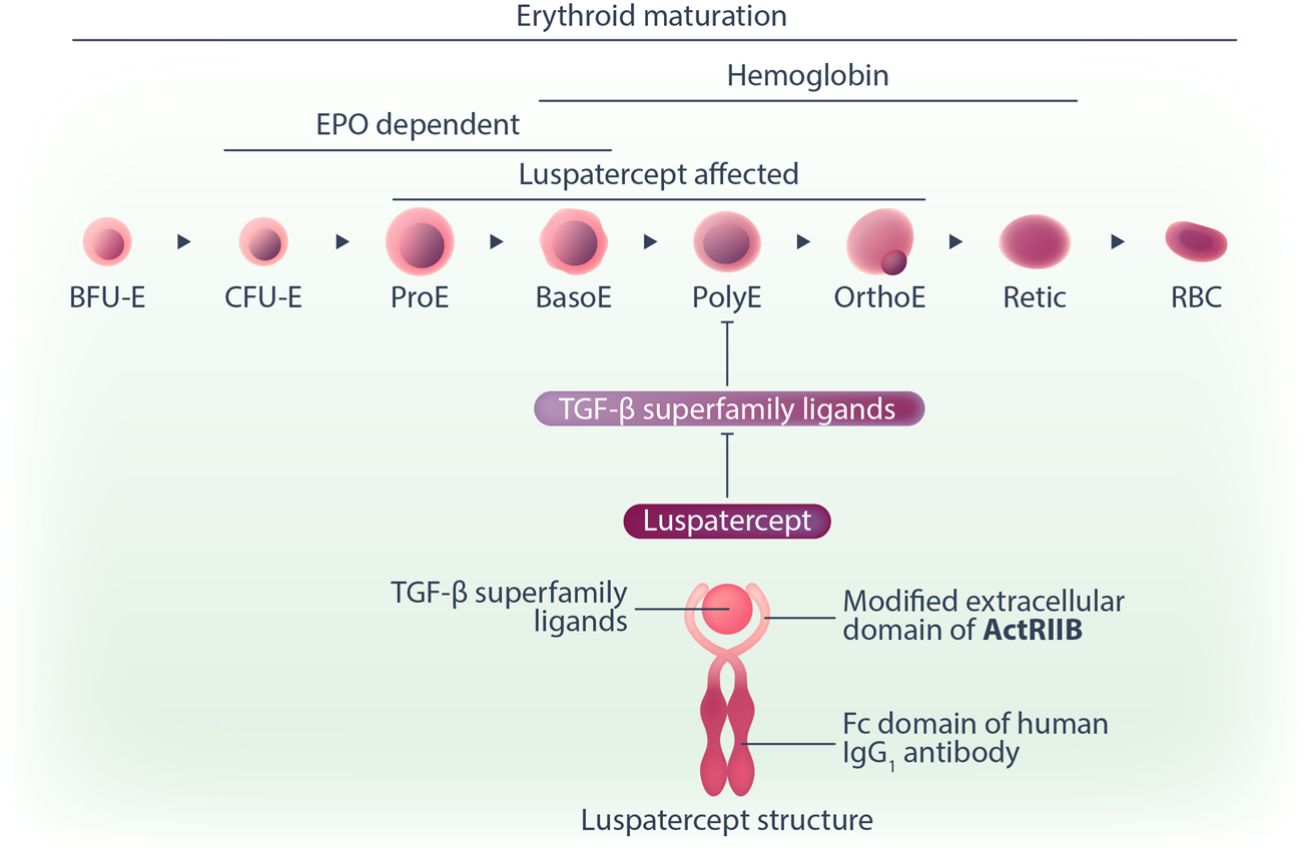
Structure and mechanism of action of luspatercept. Luspatercept is a recombinant fusion protein consisting of a modified extracellular domain of the activin receptor type IIB (ActRIIB) fused with the Fc domain of immunoglobulin G1 (IgG1). Luspatercept binds transforming growth factor‐β (TGF‐β) superfamily ligands to block their inhibitory effect on erythroid maturation in late‐stage erythroid precursors.

Importantly, luspatercept has a novel mechanism of action in that it ameliorates anemia through the improvement of late‐stage erythropoiesis, in contrast to typical ESAs, which act only on early‐stage erythropoiesis. Thus, this drug is suited for the treatment of pathologic conditions in which late‐stage erythropoiesis is defective and/or ineffective, such as MDS and β‐thalassemia.^5^

The phase II, multicenter, open‐label, dose‐finding PACE trial was the first study to investigate luspatercept clinical application in adult patients with lower‐risk MDS with anemia, with or without the need of RBC transfusions.^6^ The PACE study showed that luspatercept is effective even in patients with high EPO, regardless of prior ESA use, with a significantly higher response in patients with ring sideroblasts (RS) or SF3B1 mutations.

Because of the effectiveness and good tolerability of the drug observed in the PACE study, luspatercept was further investigated in a phase III, double‐blind, randomized, placebo‐controlled, multicenter trial. Specifically, the MEDALIST study evaluated the efficacy and safety of luspatercept versus placebo in anemic patients with RS MDS who required RBC transfusions.^7^ This trial showed that 38% of patients treated with luspatercept versus 13% treated with placebo achieved transfusion independency for 8 weeks or longer. The longest period of transfusion independence was a median 30.6 weeks in the luspatercept‐treated arm compared with 13.6 weeks in the placebo arm. After a long preclinical and clinical journey, based on the major results of the MEDALIST study, luspatercept was approved in the United States and Europe for the treatment of anemia in transfusion‐dependent adult patients with very low‐ to intermediate‐risk MDS with RS who have failed to respond to, or are ineligible for, ESA treatment.^7^, ^8^

The treatment options are very limited for low‐risk MDS patients that do not respond or lose response to ESAs and show progressive increase in RBC transfusion requirement. The COMMANDS trial, a phase III, open‐label, randomized study, was designed to compare the efficacy and safety of luspatercept with that of epoetin‐alfa for the treatment of anemia in MDS patients who require RBC transfusions.^9^ Compared with epoetin‐alfa, luspatercept led to clinically meaningful and statistically significant improvements in RBC transfusion independence and erythroid response, as well as duration of response. In particular, luspatercept improved the rate at which transfusion independence and increased hemoglobin were achieved compared with epoetin‐alfa in ESA‐naive patients with lower‐risk MDS. Nearly twice as many patients treated with luspatercept achieved transfusion independence for at least 12 weeks and concurrent hemoglobin increase compared to epoetin‐alfa (58.5% luspatercept‐treated versus 31.2% epoetin‐alfa‐treated) with a mean hemoglobin increase of at least 1.5 g/dL within the first 24 weeks. These data show, for the first time, the superiority of a novel drug over ESAs in patients with transfusion‐dependent low‐risk MDS and expanded the eligible population to ESA‐naive patients, although not regardless of RS status.^9^ Response rates in the two treatment groups were in fact similar in RS‐negative patients. Based on the results of the COMMANDS trial, luspatercept was approved by the FDA as the first‐line treatment for anemia in ESA‐naive LR‐MDS patients who require transfusion, making this drug a new standard of care for low‐risk MDS.

Through the phase II BEYOND and the phase III BELIEVE studies, effectiveness of luspatercept in improving anemia and achieving transfusion independency was also demonstrated in patients with β‐thalassemia, which was approved as an additional indication for the clinical application of this drug.^5^, ^10^

It remains to be established what TGF‐β ligands is the specific target of luspatercept and why patients with RS or SF3B1 mutation show higher response rates. Although so far no changes in the allelic burden of mutations like SF3B1 have been observed following luspatercept treatment, whether the drug has disease‐modifying activity requires further investigation in preclinical studies and future clinical trials. Finally, since ineffective/defective erythropoiesis is a feature of other pathologic conditions, the use of luspatercept should be further explored for the treatment of anemias besides MDS and β‐thalassemia.

## AUTHOR CONTRIBUTIONS

Francesca Vinchi and Uwe Platzbecker wrote the HemaTopic.

## CONFLICT OF INTEREST STATEMENT

**Francesca Vinchi:** Silence Therapeutics: Research Funding; CSL Vifor: Research Funding; PharmaNutra: Research Funding; RallyBio: Consultancy. **Uwe Platzbecker:** Janssen: Honoraria; Jazz: Honoraria; Silence Therapeutics: Honoraria; Takeda: Honoraria; Novartis: Honoraria; Abbvie: Honoraria; BMS/Celgene: Honoraria; Geron: Honoraria.

## FUNDING

This manuscript received no funding.

## Data Availability

Data sharing is not applicable to this article as no data sets were generated or analyzed during the current study.

## References

[1] Vinchi F , Hell S , Platzbecker U . Controversies on the consequences of iron overload and chelation in MDS. HemaSphere. 2020;4(3):e357. 32647792 10.1097/HS9.0000000000000357 PMC7306315

[2] Iancu‐Rubin C , Mosoyan G , Wang J , Kraus T , Sung V , Hoffman R . Stromal cell‐mediated inhibition of erythropoiesis can be attenuated by Sotatercept (ACE‐011), an activin receptor type II ligand trap. Exp Hematol. 2013;41(2):155‐166. 23261964 10.1016/j.exphem.2012.12.002

[3] Wobus M , Mies A , Asokan N , et al. Luspatercept restores SDF‐1‐mediated hematopoietic support by MDS‐derived mesenchymal stromal cells. Leukemia. 2021;35(10):2936‐2947. 34002031 10.1038/s41375-021-01275-5 PMC8478655

[4] Weidner H , Wobus M , Hofbauer LC , Rauner M , Platzbecker U . Luspatercept mitigates bone loss driven by myelodysplastic neoplasms and estrogen‐deficiency in mice. Leukemia. 2022;36(11):2715‐2718. 36175549 10.1038/s41375-022-01702-1 PMC9613459

[5] Cappellini MD , Marcon A , Fattizzo B , Motta I . Innovative treatments for rare anemias. HemaSphere. 2021;5(6):e576. 34095760 10.1097/HS9.0000000000000576 PMC8171369

[6] Platzbecker U , Germing U , Götze KS , et al. Luspatercept for the treatment of anaemia in patients with lower‐risk myelodysplastic syndromes (PACE‐MDS): a multicentre, open‐label phase 2 dose‐finding study with long‐term extension study. Lancet Oncol. 2017;18(10):1338‐1347. 28870615 10.1016/S1470-2045(17)30615-0

[7] Fenaux P , Platzbecker U , Mufti GJ , et al. Luspatercept in patients with lower‐risk myelodysplastic syndromes. N Engl J Med. 2020;382(2):140‐151. 31914241 10.1056/NEJMoa1908892

[8] Delgado J , Voltz C , Stain M , et al. The European Medicines Agency Review of luspatercept for the treatment of adult patients with transfusion‐dependent anemia caused by low‐risk myelodysplastic syndromes with ring sideroblasts or beta‐thalassemia. HemaSphere. 2021;5(8):e616. 34291195 10.1097/HS9.0000000000000616 PMC8288896

[9] Platzbecker U , Della Porta MG , Santini V , et al. Efficacy and safety of luspatercept versus epoetin alfa in erythropoiesis‐stimulating agent‐naive, transfusion‐dependent, lower‐risk myelodysplastic syndromes (COMMANDS): interim analysis of a phase 3, open‐label, randomised controlled trial. The Lancet. 2024;402(10399):373‐385. 10.1016/S0140-6736(23)00874-7 37311468

[10] Iolascon A , Rivella S , Anagnou NP , et al. The EHA Research Roadmap: anemias. HemaSphere. 2021;5(7):e607. 34522846 10.1097/HS9.0000000000000607 PMC8432644

